# Association of temperature management strategy with fever in critically ill children after out-of-hospital cardiac arrest

**DOI:** 10.3389/fped.2024.1355385

**Published:** 2024-04-10

**Authors:** Micah Kadden, Anqing Zhang, Michael Shoykhet

**Affiliations:** ^1^ Pediatric Critical Care Medicine, Children’s National Hospital, Washington, DC, United States; ^2^ Pediatric Critical Care Medicine, Ronald Reagan UCLA Medical Center, Los Angeles, CA, United States; ^3^ Division of Biostatistics and Study Methodology, Children’s National Hospital, Silver Spring, MD, United States; ^4^ Department of Pediatrics, School of Medicine and Health Sciences, The George Washington University, Washington, DC, United States

**Keywords:** post-cardiac arrest care, therapeutic temperature management, hypothermia, hyperthermia, pediatric cardiac arrest, survival, outcome, functional status scale (FSS)

## Abstract

**Objective:**

To determine whether ICU temperature management strategy is associated with fever in children with return of spontaneous circulation (ROSC) after out-of-hospital cardiac arrest (OHCA).

**Methods:**

We conducted a single-center retrospective cohort study at a quaternary Children's hospital between 1/1/2016–31/12/2020. Mechanically ventilated children (<18 y/o) admitted to Pediatric or Cardiac ICU (PICU/CICU) with ROSC after OHCA who survived at least 72 h were included. Primary exposure was initial PICU/CICU temperature management strategy of: (1) passive management; or (2) warming with an air-warming blanket; or (3) targeted temperature management with a heating/cooling (homeothermic) blanket. Primary outcome was fever (≥38°C) within 72 h of admission.

**Results:**

Over the study period, 111 children with ROSC after OHCA were admitted to PICU/CICU, received mechanical ventilation and survived at least 72 h. Median age was 31 (IQR 6–135) months, 64% (71/111) were male, and 49% (54/111) were previously healthy. Fever within 72 h of admission occurred in 51% (57/111) of patients. The choice of initial temperature management strategy was associated with occurrence of fever (*χ*^2^ = 9.36, df = 2, *p* = 0.009). Fever occurred in 60% (43/72) of patients managed passively, 45% (13/29) of patients managed with the air-warming blanket and 10% (1/10) of patients managed with the homeothermic blanket. Compared to passive management, use of homeothermic, but not of air-warming, blanket reduced fever risk [homeothermic: Risk Ratio (RR) = 0.17, 95%CI 0.03–0.69; air-warming: RR = 0.75, 95%CI 0.46–1.12]. To prevent fever in one child using a homeothermic blanket, number needed to treat (NNT) = 2.

**Conclusion:**

In critically ill children with ROSC after OHCA, ICU temperature management strategy is associated with fever. Use of a heating/cooling blanket with homeothermic feedback reduces fever incidence during post-arrest care.

## Introduction

Out-of-hospital cardiac arrest (OHCA) affects 8,000–15,000 children each year in the US alone (1–4.) Severe brain injury affects approximately 50% of long-term OHCA survivors (2, 3, 5, 6). It also contributes to the majority of short-term deaths following successful resuscitation (7, 8). No preventive or therapeutic strategy for cardiac arrest-related brain injury in children currently exists.

Early clinical trials of therapeutic hypothermia (TH) after OHCA, which allowed spontaneous thermoregulation in control cohorts, showed that fever occurred frequently in non-cooled OHCA survivors (9–12). Fever is independently associated with worse neurologic outcomes after OHCA (13–15). Modern pediatric and adult clinical trials of TH utilized active Targeted Temperature Management (TTM) in both hypo- and normothermic cohorts (16–19). These active TTM trials showed that mild hypothermia and normothermia produce similar outcomes after OHCA (16–19) (although see Harhay et al. for a Bayesian reinterpretation of the pediatric THAPCA trial 20). Current American Heart Association guidelines recommend active TTM post-OHCA, but let a clinician choose target temperature and TTM device (21, 22).

Despite the guidelines, however, TTM utilization varies across centers and may be decreasing (23, 24). Decreased TTM utilization may reflect a misperception that a normothermic temperature target implies no active temperature control. Furthermore, hypothermia on hospital admission is common among resuscitated OHCA victims (25, 26). Yet no standardized approach to rewarming exists. If associated with fever, variability in temperature management and rewarming strategies in OHCA victims may worsen brain injury (13–15, 27). We hypothesized that in critically ill children resuscitated from OHCA, ICU temperature management strategy is associated with incidence of fever. We used a single-center retrospective cohort of critically ill children with OHCA to evaluate our hypothesis.

## Methods

### Study design and IRB approval

This was a retrospective cohort study conducted within Children's National Hospital (CNH)—a large, tertiary-care pediatric hospital in Washington, DC, USA. The Institutional Review Board at CNH approved the study protocol as exempt from informed consent (#00014582). The hospital-wide electronic medical record (EMR) database (Cerner, Kansas City, MO, USA) was queried for patients with ICD-10-CM codes associated with cardiac arrest (I46.8, I46.9, G93.82, Z86.74; Supplementary Table S1) and admitted to either the Pediatric or the Cardiac Intensive Care Unit (PICU or CICU, respectively) over a 5-year period between 01/01/2016 and 12/31/2020. All subsequent information was obtained via chart review. No studies involving temperature regulation after pediatric OHCA were ongoing at CNH during the study period.

### Eligibility criteria and data abstraction

Patients ≤18 years of age were included in the study sample if they satisfied the following criteria: (1) experienced cardiac arrest, defined as absence of a pulse with unresponsiveness or perceived need for chest compressions longer than 1 min; (2) location of cardiac arrest not in a hospital and not in an emergency department; (3) OHCA associated with index admission; (4) admission to either the PICU or the CICU, (5) receiving mechanical ventilation through either an endotracheal tube or a tracheostomy on admission to the ICU; (6) not cannulated for ECMO within 72 h of admission; and (7) survived to 72 h after admission.

Patient-level data were abstracted from the medical record and included: (1) basic patient demographics (age, sex); (2) prior diagnoses; and (3) pre-arrest Functional Status Scale (FSS) (27). The pre-arrest FSS score was calculated using physician admission notes, nursing intake sheets, and, if available, prior documentation in the EMR. Abstracted CA event-level data, when available, included: (1) witnessed (yes/no); (2) bystander CPR (yes/no); (3) CPR duration; (4) number of adrenaline (epinephrine) doses administered; (5) initial ECG rhythm “shockable” (yes/no); and (6) CA cause. We did not have access to county- and city-wide EMS record systems across three states (MD, VA, WV) and the District of Columbia. Hence, variables associated with those records (e.g., time from call to EMS arrival or to CPR initiation) are not included in the analyses. Admission and hospital course data included: (1) admission (first recorded) temperature; (2) first recorded pH; (3) first recorded lactate; (4) maximum recorded temperature in the first 72 h; (5) for non-survivors, mode of in-hospital death—categorized as brain death, withdrawal of life-sustaining treatments, or CA separate from the index CA event; and (6) for survivors, discharge FSS, abstracted from physician, nursing and physical therapy documentation within 48 h of hospital discharge.

### Exposure and outcomes

The primary exposure was the temperature management strategy employed in the first 72 h of ICU admission. Temperature management interventions initiated *after* the occurrence of fever were excluded. Temperature management strategies at CNH consisted of three mutually exclusive categories: (1) passive temperature management; (2) active warming with an air-warming surface blanket without homeothermic feedback (Bair Hugger Warming System Blanket, 3M, Eden Prairie, MN, USA); or (3) homeothermic temperature management targeting a specific patient core (esophageal or rectal) body temperature with a hyper-hypothermia blanket (Blanketrol III, Gentherm Medical, Cincinnati, OH, USA). The target temperature for all groups was normothermia (36–37°C). The attending physician chose the specific temperature management method for each patient without a specific protocol in place at the time of the study. Core body temperature was measured continuously with an esophageal or rectal probe and recorded in the EMR hourly. The primary outcome was occurrence of fever (core body temperature ≥38°C) within 72 h of admission. We chose 72 h to include fever caused by inflammatory response due to post-cardiac arrest syndrome and to minimize the potential confounding effect of hospital-acquired infections. Secondary outcomes were survival to hospital discharge and increase of ≥ 3 in FSS score between admission and discharge. FSS score increase of ≥3 indicates significant new morbidity in critically-ill children (28).

### Statistical analyses

Continuous descriptors are represented as mean and standard deviation (SD) if normally distributed and as median and interquartile range (IQR) otherwise. For continuous variables, two-sample comparisons are examined using the Student's *t*-test for normally distributed data or the non-parametric Mann-Whitney test for non-normally distributed data. For two or more categorical variables, the Chi-square or Fisher Exact (FE) test is used. For comparing means across the cohorts, ANOVA or Kruskal–Wallis (KW) test is used for normally and non-normally distributed variables, respectively. If results of the ANOVA or KW analysis were significant, Tukey's or Dunn's multiple comparisons test (DMCT), respectively, was used for post-hoc comparisons between two cohorts. For testing associations, univariate and multivariate linear (least squares) or logistic regressions were used for continuous and binomial outcome variables, respectively. ANCOVA was used to compare linear regression slopes. Two-sided *α* ≤ 0.05 was considered statistically significant. Data were analyzed using SAS for Windows version 9.4 (SAS Institute Inc., Cary, NC, USA) and GraphPad Prism 8 (GraphPad Software, San Diego, CA, USA). Supplementary Figure S1 shows all relevant de-identified data.

## Results

### Descriptive characteristics

**Figure 1:**
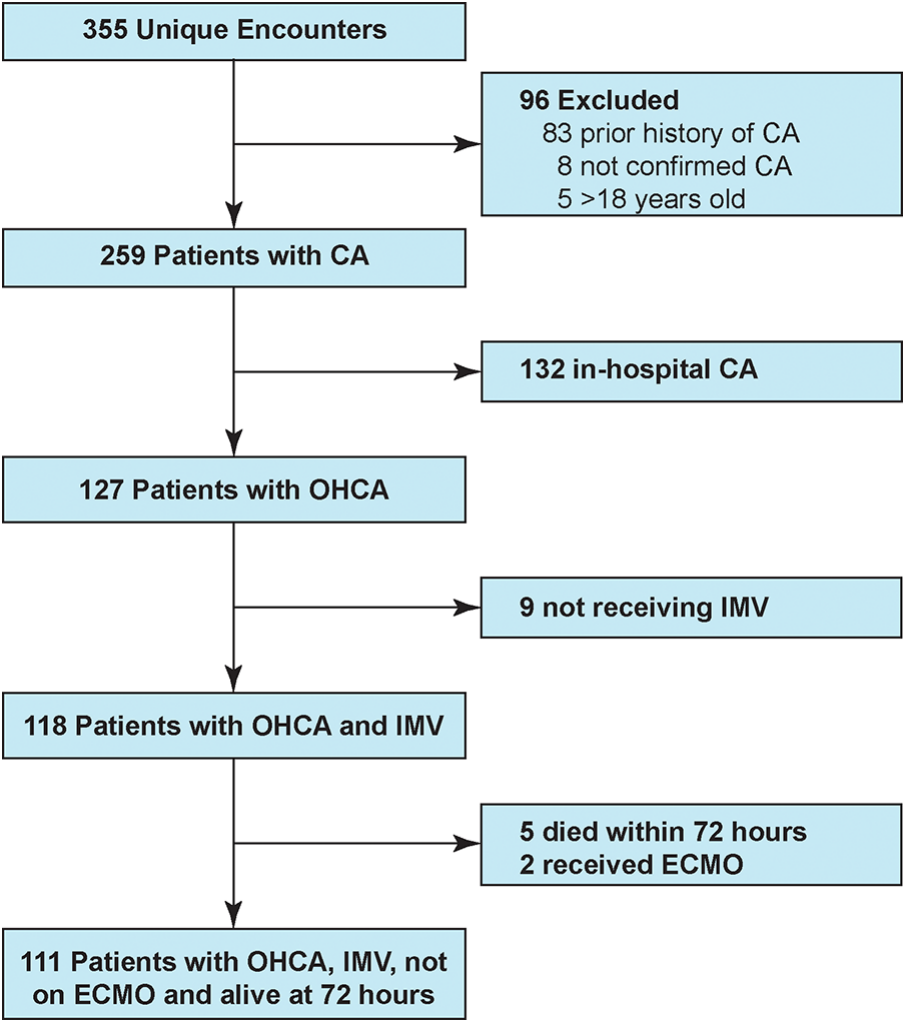
Study patient flowchart. CA, cardiac arrest; OHCA, out-of-hospital cardiac arrest; IMV, invasive mechanical ventilation.

Over the five year-long study period, CNH PICU and CICU admitted 111 mechanically ventilated children with ROSC after OHCA who survived at least 72 h (Figure 1). Table 1 shows patient demographics and arrest characteristics. Median patient age was 31 (IQR 6–135) months, 64% (71/111) were male and 49% (54/111) were previously healthy. Median pre-arrest FSS score was 6 (no disability) (IQR 6–9), with 67% (74/111) of children having no pre-existing functional disability. No child in our cohort had an admission FSS ≥ 28 (maximum FSS = 30), excluding a potential ceiling effect. Cerebral palsy was the most common pre-existing comorbidity in 16% (18/111), and 13% (14/111) of children had tracheostomies. Notably, mental health contributed to as many pediatric OHCA as congenital heart disease (each 5%).

**Table 1:** Patient demographic and cardiac arrest characteristics.

	All patients (*N* = 111)	Passive (*N* = 72)	Air-warmer (*N* = 29)	Homeothermic (*N* = 10)
General				
Age, mo	31 [6, 135]	69 [20, 144]	6 [1, 13]	95 [4, 158]
Male	71 (64)	44 (61)	21 (72)	6 (60)
Female	40 (36)	28 (39)	8 (38)	4 (40)
Pre-arrest FSS	6 [6, 9]	6 [6, 11]	6 [6, 7]	6 [6, 6]
Medical history				
Healthy	54 (49)	29 (40)	14 (47)	7 (70)
Asthma	6 (5)	5 (7)	0 (0)	1 (10)
Prior arrhythmia	4 (4)	4 (6)	0 (0)	0 (0)
Cerebral palsy	18 (16)	14 (19)	3 (10)	1 (10)
CHD	5 (5)	2 (3)	2 (7)	1 (10)
Genetic/metabolic	9 (8)	6 (8)	3 (10)	0 (0)
Mental health	6 (5)	6 (8)	0 (0)	0 (0)
Oncology	4 (4)	4 (6)	0 (0)	0 (0)
Prematurity	9 (8)	4 (6)	5 (17)	0 (0)
Tracheostomy	14 (13)	12 (17)	2 (7)	0 (0)
Arrest characteristics				
Witnessed	56 (50)	38 (53)	11 (38)	5 (50)
Bystander CPR	72 (65)	49 (68)	18 (62)	5 (50)
Witnessed with bystander CPR	40 (36)	27 (38)	11 (38)	2 (20)
CPR duration, min	19 [10, 30] *N* = 80	15 [9, 27] *N* = 53	26 [16, 37] *N* = 20	30 [17, 35] *N* = 7
Adrenaline doses	3 [1, 5] *N* = 88	2 [1, 4] *N* = 56	4 [2, 7] *N* = 25	5 [3, 6] *N* = 7
None	15 (17)	11 (21)	3 (12)	1 (11)
1	7 (8)	5 (9)	2 (8)	0 (0)
2–4	41 (47)	30 (57)	9 (36)	2 (29)
≥5	25 (28)	10 (19)	11 (44)	4 (57)
Shockable rhythm^a^	14 (13)	12 (17)	1 (3)	1 (10)
Arrest cause				
ALTE/BRUE/SIDS	12 (11)	5 (7)	5 (17)	2 (20)
Arrhythmia	7 (6)	6 (8)	0 (0)	1 (10)
Hypotension/shock	19 (17)	11 (15)	6 (21)	2 (20)
Respiratory failure	27 (24)	23 (32)	3 (10)	1 (10)
Trauma	17 (15)	5 (7)	9 (30)	3 (30)
Other	18 (16)	15 (21)	3 (10)	0 (0)
Unknown	11 (10)	7 (10)	3 (10)	1 (10)

Data are presented as median [IQR] or *N* (%). Pre-arrest FSS and prior medical history were extracted from the electronic medical record. CPR duration and number of adrenaline doses were extracted from the medical record where available, and the sample size is shown beneath the median [IQR] values. ALTE, apparent life-threatening event; BRUE, brief resolved unexplained event; CHD, congenital heart disease; FSS, functional status scale; SIDS, sudden infant death syndrome.

^a^ Initial shockable rhythm.

**Figure 2:**
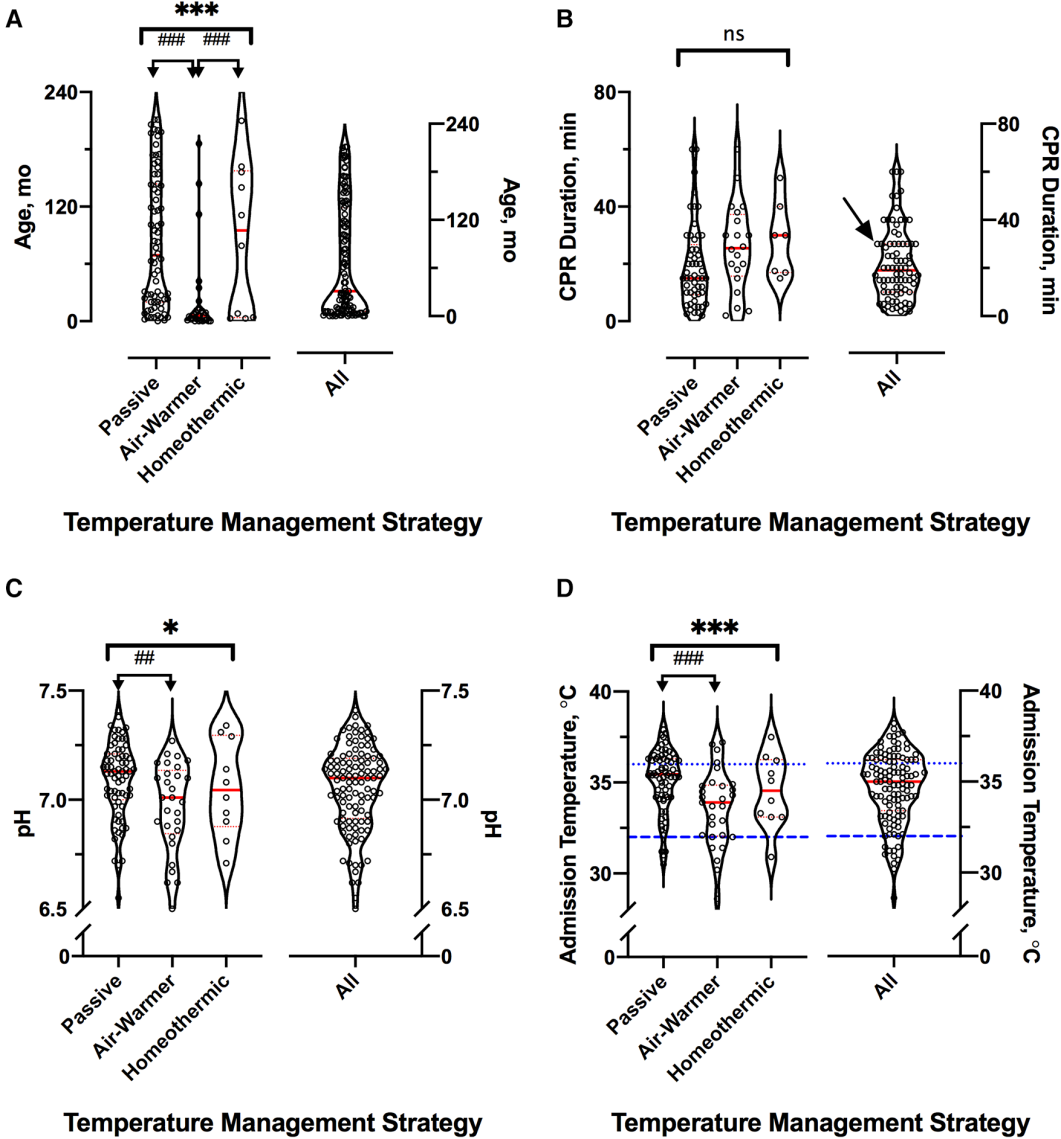
Cardiac arrest event and hospital admission characteristics. For all panels, distributions across the three temperature management cohorts are shown on the left and for the entire sample—on the right; red lines indicate median (thick) and 25th and 75th percentiles (thin). (**A**) Age distribution in months. (**B**) Reported CPR duration in minutes. Arrow at 30 min shows excess of round numbers. (**C**) First recorded whole blood pH. (**D**) Admission temperature as documented in the medical record. For clarity, dotted blue line shows the lower limit of normothermia (36°C) and interrupted blue line shows the upper limit of severe hypothermia (32°C). *represents KW analysis of variance, # represents post-hoc DMCT. Number of symbols represents *p* value: 1—*p* ≤ 0.05; 2—*p* ≤ 0.01, 3—*p* ≤ 0.001. ns, not significant.

Cohorts treated with different temperature management strategies differed in age. Children treated with an air-warming blanket were younger than children treated with either of the other two temperature management strategies (KW statistic 25.4, *p* < 0.001; DMCT *air-warmer* vs. *passive* adjusted *p* < 0.001, *air-warmer* vs. *homeothermic* adjusted *p* = 0.025, Figure 2A). Except age, other baseline characteristics were similar among the cohorts.

Arrest event characteristics (Table 1) were comparable to those described in prior studies of pediatric OHCA (1–3, 5, 7, 8). Overall, 50% (56/111) of arrests were witnessed, and 65% (72/111) of children received bystander CPR. Respiratory failure was the most common OHCA cause (24%, 27/111), followed by shock/hypotension (17%, 19/111) and traumatic arrest (15%, 17/111). Initial shockable rhythm was found in 13% (14/111) of patients. The median reported CPR duration was 19 min [IQR 10–30]. Reported CPR duration was longer among children eventually treated with either the air-warming [median 26 (IQR 16–37) min] or the homeothermic [median 30 (IQR 17–40) min] blanket than among those treated passively [median 15 (IQR 9–27) min], but the difference did not reach statistical significance (Figure 2B, KW statistic 5.96, *p* = 0.051). However, our sample of reported CPR durations had an excess of round numbers, especially at longer arrest durations, which suggests presence of recall bias (Figure 2B). The remainder of arrest characteristics also did not differ among the temperature management cohorts.

In contrast, admission characteristics clearly differed among the cohorts with respect to first recorded values of pH, lactate and temperature (T_adm_), all measured on arrival to the hospital (Table 2; Figure 2C). Compared to patients eventually treated passively, patients eventually managed with the air-warming blanket had lower first documented pH (Figure 2C; KW statistic 7.54, *p* = 0.023; DMCT *air-warmer* vs. *passive* adjusted *p* = 0.020). Their serum lactate was also higher, although on post-hoc analysis the difference did not reach statistical significance (not shown; KW statistic 7.10, *p* = 0.029; DMCT *air-warmer* vs. *passive* adjusted *p* = 0.08). Similarly, T_adm_ was lower in patients treated with the warmer than in those treated passively (Figure 2D; KW statistic 12.7, *p* = 0.002; DMCT *air-warmer* vs. *passive* adjusted *p* = 0.001). Unlike patients eventually treated with the air-warming blanket, patients eventually treated with the homeothermic blanket did not differ in pH, lactate or T_adm_ from patients treated with the passive temperature management strategy (DMCT *homeothermic* vs. *passive* adjusted p's = 1.0, 0.94 and 0.75, respectively).

**Table 2:** Hospital admission characteristics of children with ROSC after OHCA.

	All patients (*N* = 111)	Passive (*N* = 72)	Air-warmer (*N* = 29)	Homeothermic (*N* = 10)	*p* value (test)
Admission characteristics					
First pH *N*	7.10 [6.9, 7.2] 108	7.13 [7.0, 7.2] 69	7.01 [6.8, 7.1] 29	7.05 [6.9, 7.3] 10	**0.02** (KW)
Lactate, mg/dl *N*	7.5 [3.7, 12] 105	7.0 [3.3, 11] 67	10 [5.8, 13] 28	5.1 [1.8, 9] 10	**0.03** (KW)
Tadm, °C	34.7 (±1.94)	35.2 (±1.71)	33.6 (±2.09)	34.5 (±1.98)	< **0.01** (ANOVA)
Admission hypothermia (Tadm ≤ 36 °C)	78 (70)	46 (64)	25 (86)	7 (70)	0.09 (*χ*^2^)
Severe (Tadm ≤ 32 °C)	12 (11)	4 (6)	7 (24)	1 (10)	**0.03** (FE)

Data are presented as median [IQR], mean (±SD) or number (%). Blood pH and lactate values are from first documented in-hospital whole blood gas analysis. T_adm_ = first recorded core body temperature, KW, Kruskal–Wallis; FE, fisher exact; ECMO, extracorporeal membrane oxygenation.

Bold values means the statistically significant values.

### Primary outcome

**Figure 3:**
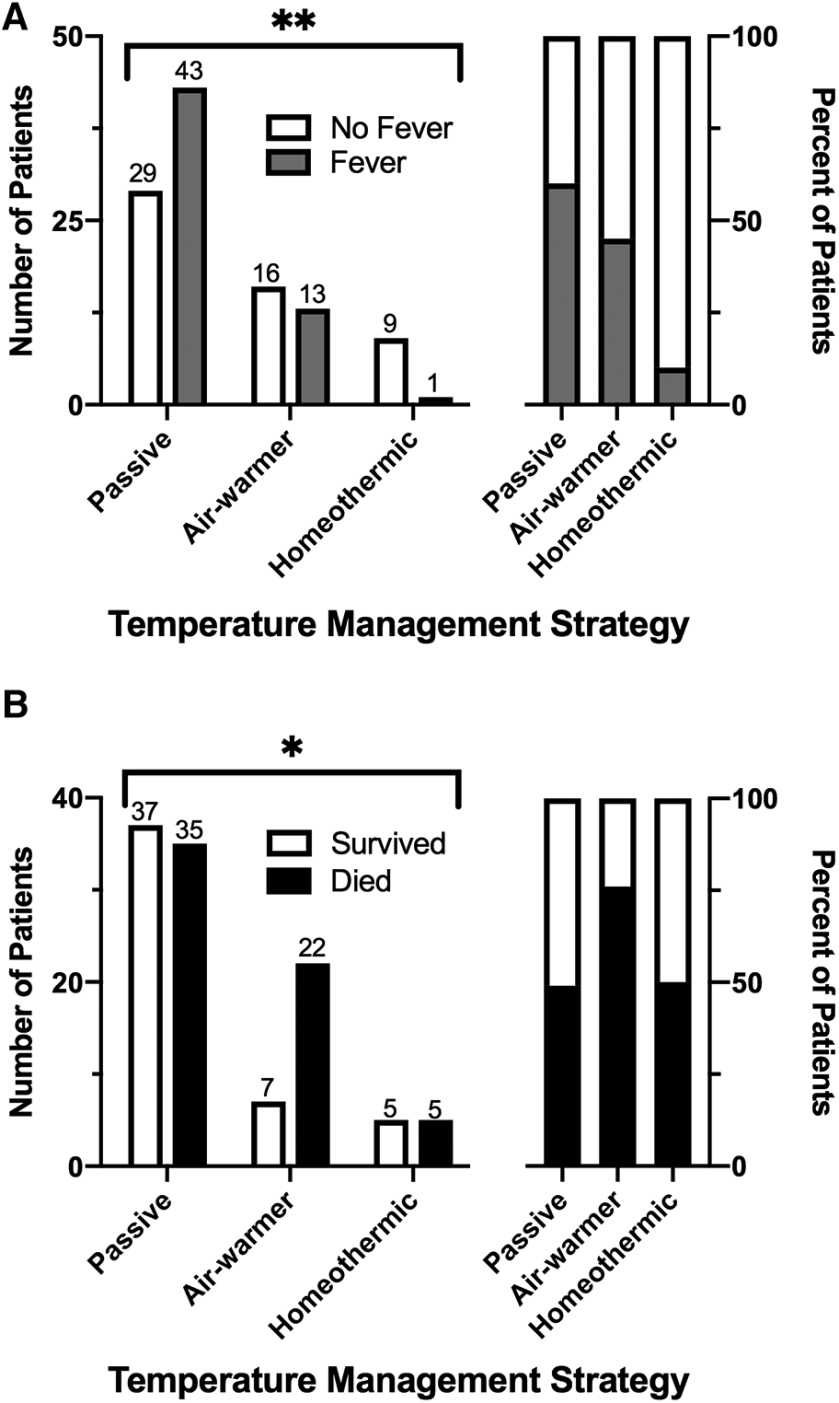
Association of temperature management strategy with fever and survival after pediatric OHCA. (**A**) Incidence of fever across the temperature management cohorts expressed as absolute number (left) and as percent (right) of patients. (**B**) Survival to hospital discharge across the temperature management cohorts, expressed as in (**A**). *χ*^2^, *—*p* ≤ 0.05, **—*p* ≤ 0.01.

We first examined whether initial temperature management strategy is associated with the primary outcome—subsequent fever. Overall, fever within 72 h of admission occurred in 51% (57/111) of patients. Fever occurred in 60% (43/72) of patients managed passively, 45% (13/29) of patients managed with the air-warming blanket and 10% (1/10) of patients managed with the homeothermic blanket (Figure 3A). The choice of initial temperature management strategy was associated with occurrence of fever (*χ*^2^ = 9.36, df = 2, *p* = 0.009). Compared with passive temperature management, early use of the homeothermic blanket was associated with decreased risk of having a fever within 72 h of admission (homeothermic blanket: RR = 0.17, 95% CI 0.03–0.69), whereas use of the air-warming blanket was not (air-warming blanket: RR = 0.75, 95% CI 0.46–1.12) (Table 3).

**Table 3:** Association of temperature management strategy with outcomes after pediatric OHCA.

Outcomes	All patients (*N* = 111)	Passive (*N* = 72)	Air-warmer (*N* = 29)	Homeothermic (*N* = 10)	*p* value (test)
Primary					
Fever	57 (51)	43 (60)	13 (45)	1 (10)	<**0.01** (*χ*^2^)
Relative risk, (95%CI)		Ref	0.75[0.46–1.12]	**0.17 [0.03–0.69]**	
Secondary					
Survived (hosp)	49 (44)	37 (51)	7 (24)	5 (50)	**0.04** (*χ*^2^)
Survived (28 d)	46 (41)	36 (50)	6 (21)	4 (40)	
Discharge FSS	10 [7, 23]	10 [7, 23]	9 [6, 25]	10 [7, 21]	0.90 (KW)
*Δ*FSS ≥ 3	15 (31)	10 (27)	3 (43)	2 (40)	0.51 (FE)

Data are presented as median [IQR] or number (%). *Δ*FSS is change in FSS score between admission and discharge. KW, Kruskal–Wallis; FE, fisher exact; FSS, functional status scale.

Bold values means the statistically significant values.

Since the cohorts treated with different temperature management strategies differed in several respects (e.g., age), we conducted univariate analyses to determine which additional variables were potentially associated with fever (Table 4). Variables with *p* ≤ 0.1 on univariate analyses were then included in a multivariate logistic regression with fever as outcome. Of several potentially confounding variables, only age and temperature management strategy were associated with fever on univariate analyses (Table 4, unadjusted *p* = 0.10 and <0.01, respectively). When these two variables were included in multivariate logistic regression, only temperature management strategy remained significantly associated with incidence of fever (Table 4, adjusted *p* < 0.01).

**Table 4:** Univariate and multivariate analyses of variables associated with fever.

Logistic Regression	β	SE	95% CI	*p* value
Univariate				Unadjusted
Age	0.004	0.003	[−0.001, 0.001]	**0.10**
First pH	−0.518	1.006	[−2.531, 1.447]	0.60
CPR Duration	−0.015	0.016	[−0.047, 0.015]	0.33
Tadm	−0.008	0.098	[−0.203, 0.186]	0.94
Temperature strategy	−0.973	0.331	[−1.662, −0.354]	<**0.01**
Multivariate				Adjusted
Age	0.004	0.004	[0.004, 0.0118]	0.34
Temperature strategy	−1.036	0.426	[−1.944, −0.241]	**0.01**

Association between variables and fever was examined using logistic regression. Variables with unadjusted *p* ≤ **0.1** on univariate analysis were included in the multivariate analysis.

### Secondary outcomes

We next considered whether initial temperature management strategy is associated with predefined secondary outcomes—survival to hospital discharge and increase in functional disability as measured by *Δ*FSS ≥ 3. Overall, 44% (49/111) of patients survived to hospital discharge and 41% (46/111) survived to 28 days (Table 3). As expected from other studies of pediatric OHCA (5, 7, 8), overall survival in our cohort was associated with presenting rhythm (shockable vs. non-shockable), number of adrenaline doses during resuscitation, CPR duration, and severity of acidemia and hyperlactatemia on the first in-hospital blood gas (Supplementary Figure S2). Choice of temperature management strategy was also associated with survival (Figure 3B, *χ*^2^ = 6.38, df = 2, *p* = 0.041). Lowest survival to hospital discharge (24%, 7/29) and at 28 days after discharge (21%, 6/29) occurred among children treated with the air-warming blanket. Since multiple factors impact OHCA survival, we used multivariate logistic regression to control for potential confounding variables. With these variables included in the analysis, temperature management strategy was not independently associated with survival (Supplementary Table S2).

Functional disability overall was more prevalent in survivors at discharge, reflected by a 4 point increase in the median FSS score from admission (Table 3, MW, *p* < 0.001). Among temperature management cohorts, however, median discharge FSS was similar (Table 3, KW statistic 0.21, *p* = 0.90). The proportion of surviving patients with *Δ*FSS ≥ 3 also did not differ among cohorts (Table 3, FE, *p* = 0.51).

### Temperature management strategy and temperature trends over time

**Figure 4:**
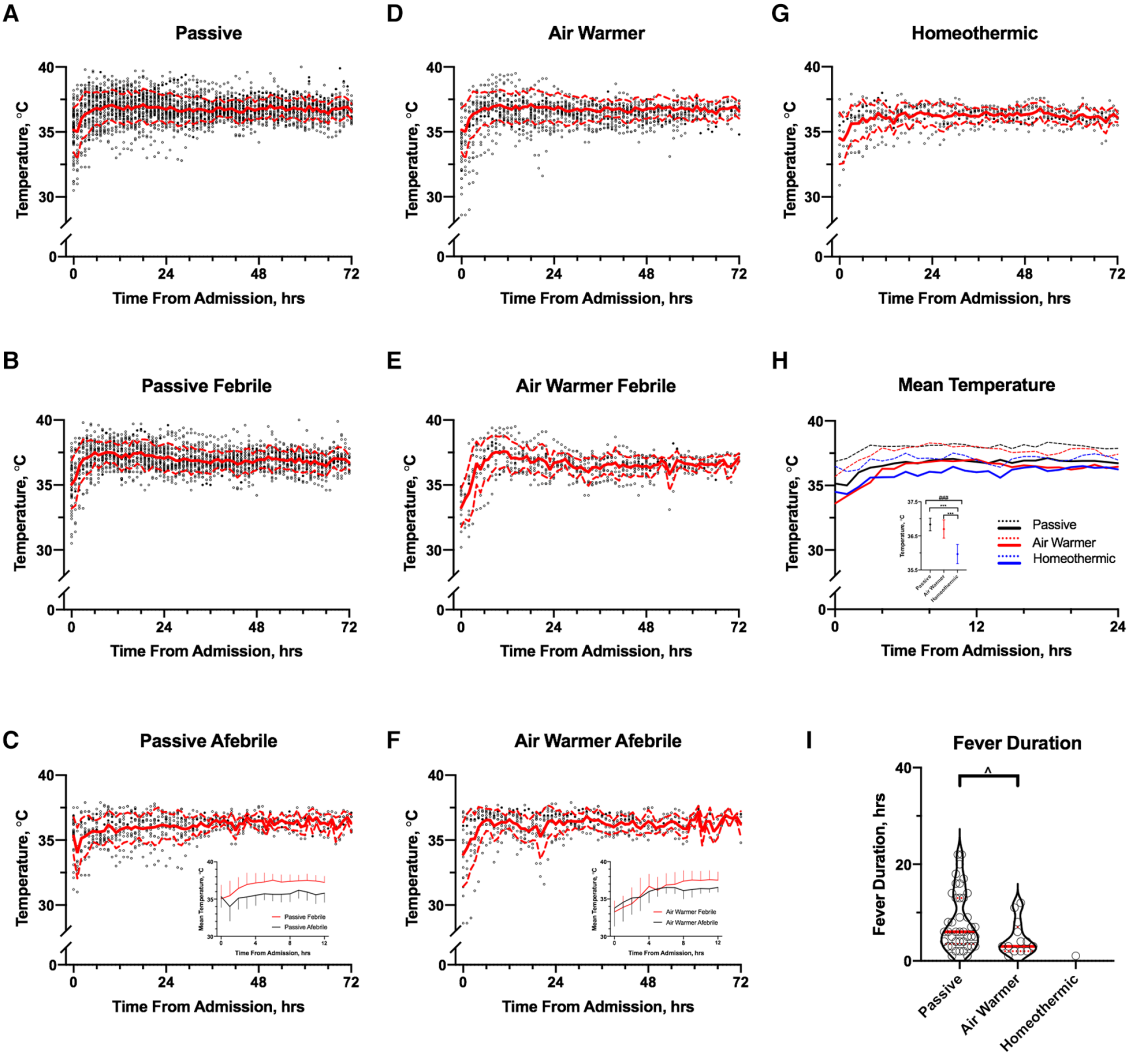
Association of temperature management strategy with temporal temperature trends. **A**–**C** Temperature trends over 72 h from admission in all (**A**), febrile (**B**) and afebrile (**C**) passively managed patients; (**C**) **inset**: comparison of mean (±SD) temperatures of febrile (red) and afebrile (black) passively managed patients over the first 12 h. (**D**–**F**) Temperature trends over 72 h from admission in all (**D**), febrile (**E**) and afebrile (**F**) patients managed with the air-warming blanket; (**F**) **inset**: comparison of mean (±SD) temperatures of febrile (red) and afebrile (black) patients managed with the air-warming blanket over the first 12 h. (**G**) Temperature trends over 72 h from admission in all patients managed with a homeothermic blanket. In A-G, mean and SD are shown by solid and dashed red lines, respectively. (**H**) Comparison of mean temperatures in the first 24 h after admission among the cohorts managed with the 3 temperature management strategies. Dashed lines show SD; (**H**) **inset**: comparison of mean temperatures averaged over 12 h by temperature management strategy. Whiskers show SD. # ANOVA *p* < 0.01, *Tukey's MCT *p* < 0.01. (**I**) Association of fever duration with temperature management strategy. One observation (36 h) in the passive group omitted from graph for clarity but included in the analysis. Solid and dashed red lines show median and 25–75th percentiles, respectively. ^MW, *p* = 0.03.

Figure 4 shows temperature as a function of time from admission in patients stratified by the temperature management strategy. The mean temperature in cohorts managed passively or with an air-warming blanket (Figures 4A,D, respectively) changed faster in the 1st 12 h than in the subsequent 60 h. In patients managed with a homeothermic blanket, initial increase in mean temperature occurred over a slower interval of ∼24 h (Figure 4G). Use of the homeothermic blanket significantly lowered mean temperature during the 1st 12 h after admission (Figure 4H) by ∼1°C compared to either passive management or use of the air-warming blanket (Figure 4H inset; ANOVA *p* < 0.001, followed by Tukey's MCT).

When patients in the passive (Figures 4B,C) and air-warming cohorts (Figures 4E,F) are stratified by fever status, several observations emerge (homeothermic cohort is omitted because only a single observation of T = 38°C occurred). First, mean admission temperatures did not differ between febrile and afebrile patients in either cohort (data not shown). Second, in both cohorts mean temperature increased faster in the first 12 h in febrile patients than in afebrile patients (linear regression slope [95% CI] in °C/h; passive: febrile 0.20 [0.16, 0.24], afebrile 0.09 [0.03, 0.14], ANCOVA for difference between slopes *p* = 0.001; air-warmer: febrile 0.37 [0.30, 0.45], afebrile 0.20 [0.14, 0.27], ANCOVA for difference between slopes *p* = 0.001). In the passive cohort, a clinically meaningful difference (∼1°C) in mean temperature between febrile and afebrile patients occurred within the 1st hour after admission (Figure 4C inset). In the air-warming cohort, this difference occurred later at ∼6 h after admission (Figure 4F inset). Temperature management strategy also correlated with fever duration (Figure 4I). Fevers lasted longer in the passive cohort than in the air-warming cohort (median [95% CI]; passive 6 [4, 9] h, air warmer 3 [2, 8] h; MW *p* = 0.03). Notably, only a single observation of T = 38°C for one hour occurred in the homeothermic cohort.

### Antipyretic Use

We considered if antipyretic use could have influenced the occurrence of fever in the different temperature management cohorts. Specifically, we asked whether antipyretic use as pretreatment of fever and/or its median daily dose in pretreated patients were different among the cohorts. For this analysis, we defined patients as “pretreated” if (1) they received an antipyretic before the occurrence of fever or (2) they received an antipyretic and never developed a fever. The proportion of pretreated patients did not differ among the temperature management cohorts (35% (25/72), 24% (7/29) and 30% (3/10) for passive, air-warmer and homeothermic, respectively; *χ*^2^ = 1.08, df = 2, *p* = 0.58). Acetaminophen was the first line antipyretic medication in all but one patient, who was pretreated with ketorolac. The median daily weight-based dose of acetaminophen in pretreated patients did not differ among the cohorts (KW statistic = 5.71, *p* = 0.06). These data suggest that acetaminophen use did not systematically vary across the temperature management cohorts.

### Exploratory analyses

**Figure 5:**
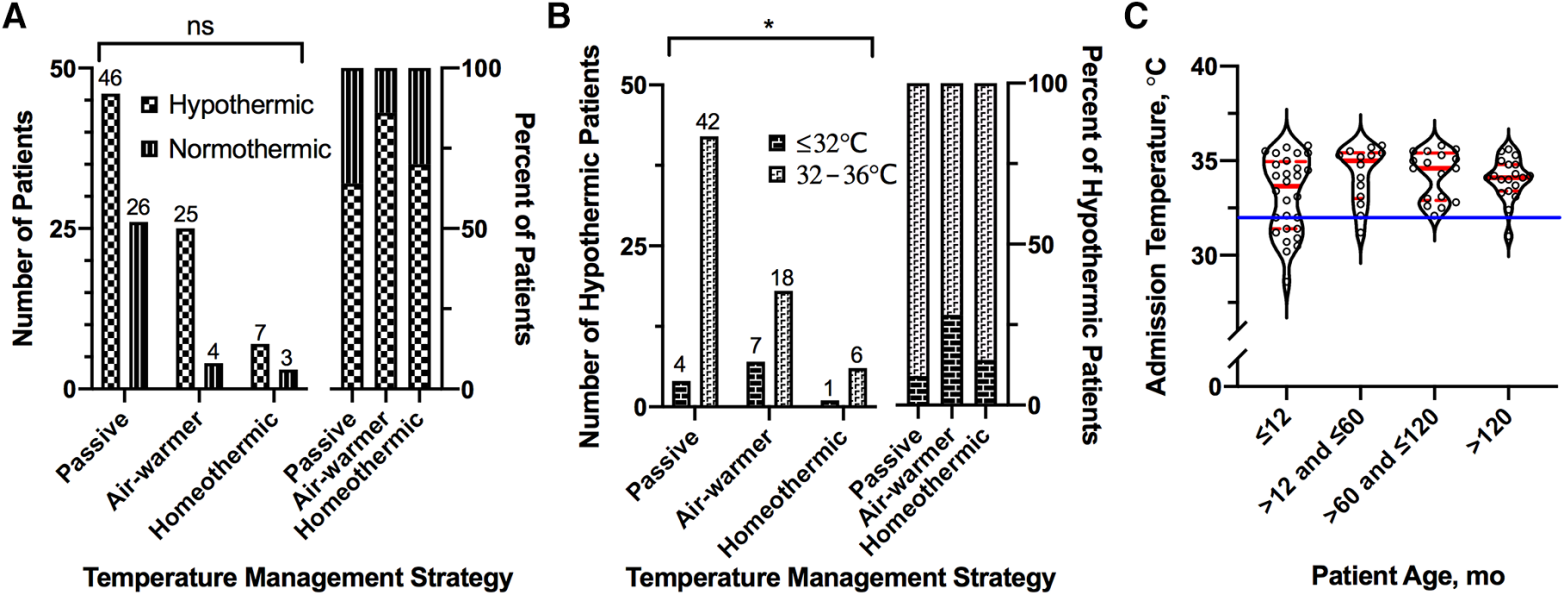
Association of admission hypothermia with temperature management strategy and age. (**A**) Number (left) and percent (right) of patients presenting with hypothermia (<36°C) on admission, stratified by temperature management strategy. (**B**) Number (left) and percent (right) of hypothermic patients presenting with moderate-to-severe hypothermia (≤32°C), stratified by temperature management strategy. (**C**) Distribution of admission temperatures among patients presenting with hypothermia, stratified by age. Solid blue line shows 32°C. For all violin plots, thick and thin red lines represent median and 25th/75th percentiles, respectively. For bar graphs, numbers above bars represent actual number of patients in the group. *Fisher Exact, *p* ≤ 0.05.

Given the observed variation in core body temperature on admission (see above), we considered how admission hypothermia relates to the subsequent choice of ICU temperature management strategy. Hypothermia on admission (core body temperature <36°C) occurred in 70% (78/111) of patients (Figure 5). Overall frequency of hypothermia on admission was similar across the temperature management cohorts (Figure 5A, *χ*^2^ = 4.93, df = 2, *p* = 0.09). Moderate-to-severe hypothermia (≤32°C), however, did differ across the cohorts (FE, *p* = 0.03); it occurred more frequently in the group eventually treated with the air-warming blanket (24%, 7/29) than in the groups treated passively (5%, 4/72) or with the homeothermic blanket (10%, 1/10; Figure 5B).

The majority of instances of severe hypothermia (83%, 10/12) occurred in infants 12 months of age or younger (Figure 5C). Across the entire sample, however, age was not associated with admission temperature (simple linear regression, slope [95% CI] = 0.003 [−0.003, 0.008], *p* = 0.33). Almost ¾ of these severely hypothermic infants (70%, 7/10) were treated with the air-warming blanket. Similarly, infants (≤12 months of age) with any degree of hypothermia on admission (*n* = 28) were twice as likely to be treated with the air-warming blanket (64%, 18/28) than with passive management (32%, 9/28). In contrast, only 14% (7/50) of children older than 12 months of age with hypothermia on admission were treated with the air-warming blanket. These data suggest that among hypothermic OHCA victims, the air-warming blanket was preferentially deployed in infants.

## Discussion

In this single-center retrospective cohort study of children admitted to intensive care after resuscitation from OHCA, choice of PICU/CICU temperature management strategy was associated with post-arrest fever. Compared to passive temperature management, proactive use of a homeothermic temperature control blanket, but not of an air-warming blanket, was associated with reduced risk of fever within 72 h of admission. With the passive temperature management group as the reference, two children would need to be treated with a homeothermic blanket to prevent occurrence of fever in one child (Absolute Risk Reduction (ARR) = 0.6–0.1 = 0.5, Number-Needed-to-Treat (NNT) = 1/ARR = 2).

The analysis also revealed a weak association between choice of temperature management strategy and survival to hospital discharge. Specifically, children treated with the air warming blanket had the lowest survival probability. The association was not significant when other variables influencing OHCA survival were included in multivariate analysis. The latter finding indicates that factors other than choice of temperature management strategy likely mediate this association. Children treated with the air-warming blanket appear to constitute a subgroup of children with ROSC after OHCA. This subgroup is characterized by age ≤12 months, higher frequency of severe hypothermia on admission, lower initial serum pH, higher initial serum lactate and lower survival probability. We propose that the observed association between choice of temperature management and survival to hospital discharge is mediated by arrest etiologies peculiar to this subgroup—non-accidental trauma and BRUE/SIDS—and traditionally associated with poor outcomes (5, 7, 8). These factors may require consideration when designing future pediatric OHCA trials.

Our study has limitations. The primary limitation is that the number of patients treated with the homeothermic blanket is small, opening the study to selection and temporal biases. Hence, our findings require confirmation in a larger and multi-institutional cohort. As a retrospective cohort study, it allows for assessment of associations, not of causality. For example, we did not have access to detailed pre-hospital EMS records, and may have missed important contributing variables. Factors other than antipyretic use may have influenced occurrence of fever in our sample. It is possible that occurrence of fever in the post-arrest period is associated with injury severity and related inflammatory state. This study's relatively small sample size does not allow for adequate propensity matching to investigate the interaction among injury severity, fever occurrence and choice of temperature management strategy. We did not investigate the occurrence of fever beyond initial 72 h in the ICU, and later fevers may also impact outcome.

Our single-center patient sample appears representative of the population of children who experience OHCA. The findings that survival to hospital discharge is associated with shockable initial rhythm, shorter CPR duration and fewer epinephrine doses are comparable to those in previous studies on OHCA (3, 5, 7, 8). Similarly, lower lactate and higher pH on admission have also been associated with OHCA survival (5). Furthermore, distribution of arrest etiologies and overall survival are also similar to previously reported cohorts (3, 5, 7, 8). Based on these similarities, our findings may generalize to children with ROSC after OHCA at other pediatric centers.

Analyses of temperature trends over time revealed several findings of potential clinical importance. First, most salient temperature changes in post-OHCA patients occur in the first 12–24 h after admission. Thus, particular focus on temperature management after OHCA likely needs to occur as soon as the patient arrives in the ICU. Second, use of a homeothermic blanket starting at admission lowers mean temperature by a clinically meaningful 1°C in the first 12 h after admission. TTM in post-OHCA patients with a device that integrates homeothermic feedback and targets a set body temperature of 35–36°C may safely limit excursions above 38°C. Third, core body temperature rises faster in febrile than in afebrile patients in the first 12 h after admission. In resource-limited settings where TTM with a homeothermic device is unavailable, attention to rate of temperature increase early after admission may help identify post-OHCA patients at risk of fever. Finally, while air-warming blanket may not decrease fever incidence compared to passive management, it may decrease fever “dose” by shortening its duration.

Hypothermia on admission occurred in 70% of children in our sample, similar to prior studies in OHCA (25, 26). High prevalence of hypothermia likely explains why 35% of our patients were actively warmed on admission with either a homeothermic or an air-warming blanket. The perceived need to achieve euthermia relates to potential risks of hypothermia—arrhythmia, coagulopathy, and immune dysfunction (29–34). However, prior pediatric studies have not shown increased incidence of serious arrhythmias, clinically significant bleeding, or increased infections in pediatric patients treated with core temperatures of 32–34°C compared to normothermic patients (18, 19). Hence, normalization of body temperature in mildly hypothermic (32–36°C) OHCA patients rarely requires urgency. Instead, OHCA patients may benefit from a systematic approach to achieve target temperature while minimizing fever risk. Our data suggest that fever in children resuscitated from OHCA is least likely when a homeothermic temperature control blanket is deployed proactively regardless of admission temperature.

While the frequency of hypothermia did not differ among the temperature management cohorts, its severity did. Severe hypothermia (≤32°C) was more common in patients treated with the air-warming blanket than in the other two treatment groups. The effect appears to be mediated by age, since 83% of children presenting with severe hypothermia were infants younger than 12 months of age. Therefore, at least in our institution, the practice pattern favors deployment of the air-warming blanket in hypothermic infants after ROSC. Possible drivers of this pattern include ease of deployment of air-warming vs. homeothermic blanket as well as availability of age-appropriate cooling pads. Use of an air-warming blanket may contribute to incidence of fever in this population, as rebound hyperthermia is common after rewarming from hypothermia (35). These data suggest that temperature management in infants resuscitated from OHCA requires particular attention in the prehospital setting.

Current American Heart Association (AHA) guidelines recommend universal TTM utilization in pediatric (21, 22) and adult (36) OHCA survivors, with the choice of hypothermia or normothermia as the target temperature left to the clinician. Despite these recommendations, TTM use in the real world is far from universal. Even shortly after completion of pediatric TTM trials (18, 19), 15% of practitioners did not choose TTM for simulated cases (37). In a retrospective analysis of adult OHCA patients who were unconscious at hospital admission, 42% did not receive TTM (38). In a recent National Inpatient Sample data set from 2016 to 2019, only 0.85% of adult OHCA survivors received TTM, and TTM utilization varied significantly by age, sex, hospital type and by region (39). In addition, methods of implementing TTM vary across providers, with one single-center study documenting 14 unique temperature management interventions among 84 patients (40). Consistent with the gap between theoretical recommendation and practical implementation, 65% of patients in our sample received no proactive temperature management. These passively managed patients also had the highest incidence of fever. Our data indicate that, in accordance with AHA Guidelines, early TTM should be routinely incorporated into post-arrest care in children. Currently commercially available non-invasive TTM devices in pediatrics include water-circulating systems as one used in this study and gel-based systems. Future studies will need to evaluate which homeothermic temperature control systems best achieve and maintain TTM after OHCA in children.

## Conclusion

Children resuscitated from OHCA frequently experience temperature dysregulation. Hypothermia on admission and fever within 72 h of admission are both common. Current data do not support passive temperature management or an air-warming blanket as effective TTM methods. Proactive TTM strategy with a heating/cooling blanket with built-in homeothermic feedback may reduce incidence of post-arrest fever. Future studies need to evaluate how specific TTM strategies impact outcome in children after OHCA.

## Data Availability

The original contributions presented in the study are included in the article/**Supplementary Material**, further inquiries can be directed to the corresponding author.

## Supplementary material

Supplementary Figure S1 De-identified data. Data are shown in reverse age order to prevent inference of admission dates. Relevant codes are within the Title Row.

Supplementary Figure S2 Variables Associated with Survival to Hospital Discharge Among Children with ROSC after OHCA. (**A**) Association between presenting rhythm and survival. (**B**) Association between the number of adrenaline doses and survival. (**C**) Association between reported CPR duration and survival. Association between pH (**D**) and lactate (**E**) values on first in-hospital whole blood gas and survival. Numbers above bars in (**A**,**B**) show sample size. Red lines in (**C**–**E**) show median (thick) and 25th and 75th percentiles (thin). ***χ*^2^ *p* ≤ 0.01; ^##^Fisher Exact, *p* ≤ 0.01; ^^^Mann–Whitney, *p* ≤ 0.001.

Supplementary Table S1 ICD-10-CM codes used to identify patients for chart review. ICD-10-CM—International Classification of Diseases, Tenth Revision, Clinical Modification.

Supplementary Table S2 Multivariate Logistic Regression of Variables Associated with Survival After OHCA. Variables with *p* ≤ 0.1 on univariate logistic regression were included in the multivariate analysis with several exceptions to account for variable covariance (e.g., lactate and pH). Significant *p* values are bolded. P, passive; AW, air-warming blanket; H, homeothermic blanket; Epi, epinephrine.
