# Supplementary figures and images for: Association of temperature management strategy with fever in critically ill children after out-of-hospital cardiac arrest
Source: Front Pediatr. 2024 Apr 10;12:1355385. doi: 10.3389/fped.2024.1355385 (PMC11039828; doi:10.3389/fped.2024.1355385)

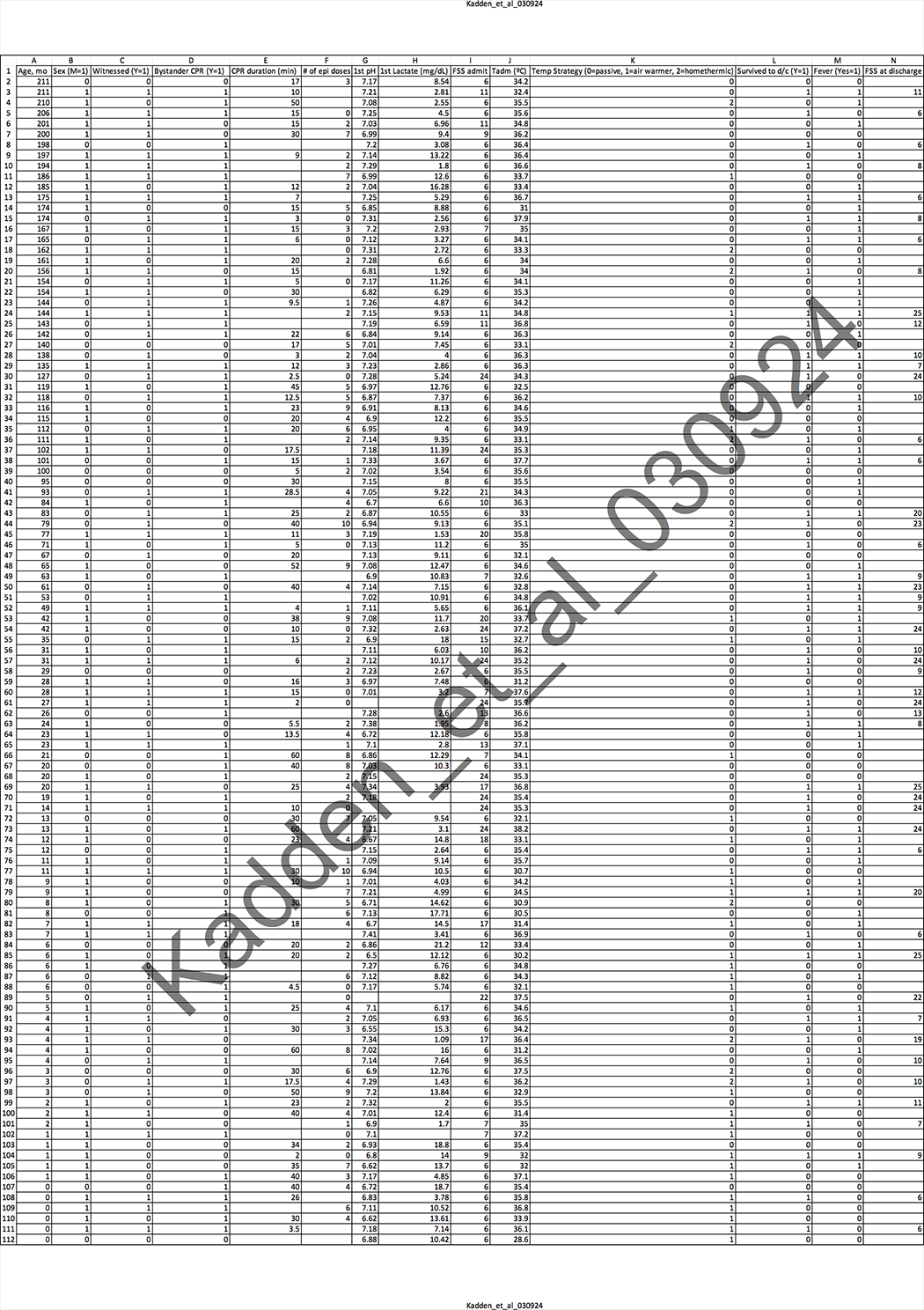

Supplement: Supplementary Figure S1 — De-identified data. Data are shown in reverse age order to prevent inference of admission dates. Relevant codes are within the Title Row. [file Image1.tif]

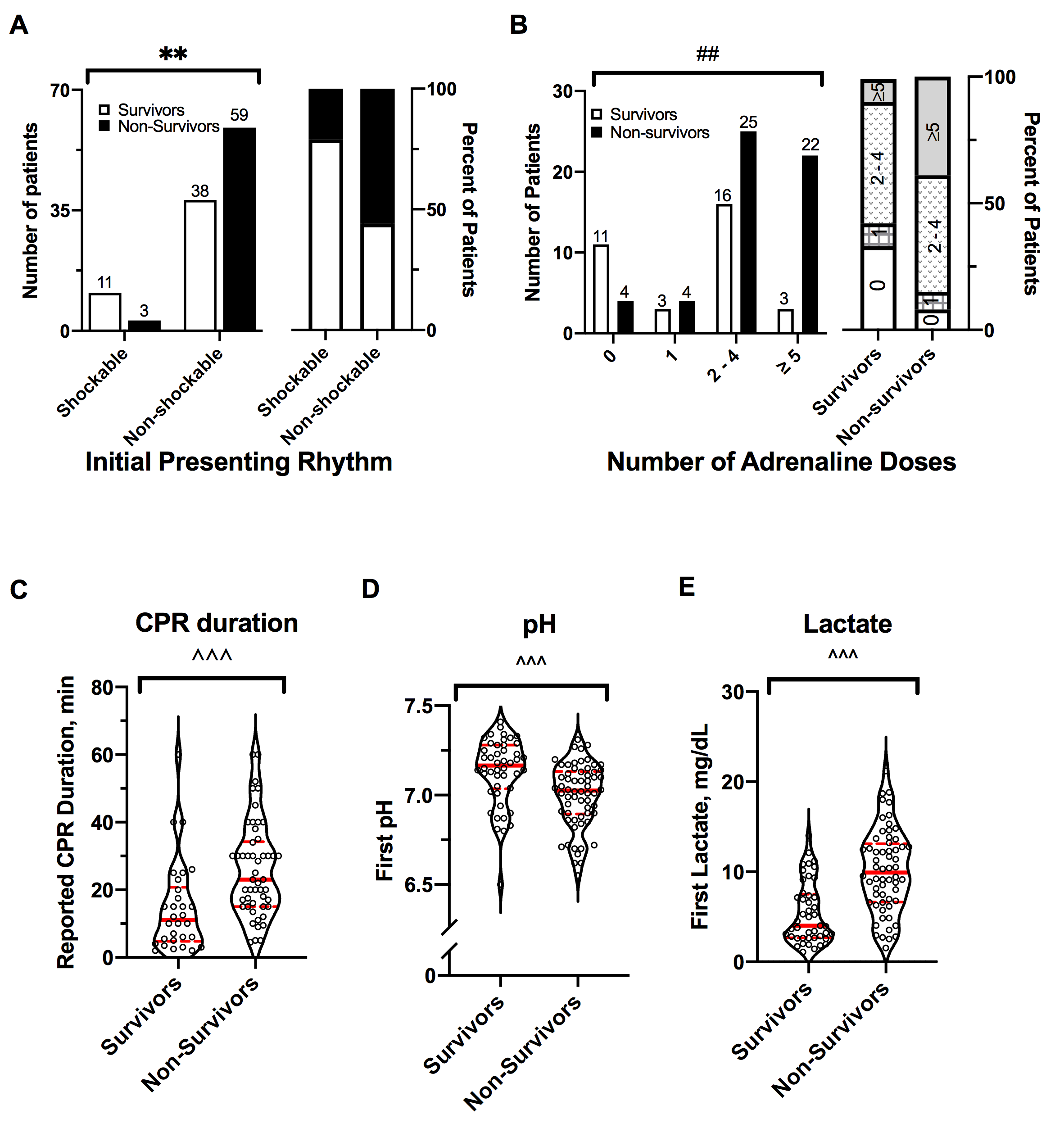

Supplement: Supplementary Figure S2 — Variables Associated with Survival to Hospital Discharge Among Children with ROSC after OHCA. (A) Association between presenting rhythm and survival. (B) Association between the number of adrenaline doses and survival. (C) Association between reported CPR duration and survival. Association between pH (D) and lactate (E) values on first in-hospital whole blood gas and survival. Numbers above bars in (A,B) show sample size. Red lines in (C–E) show median (thick) and 25th and 75th percentiles (thin). **χ2 p ≤ 0.01; ##Fisher Exact, p ≤ 0.01; ^^^Mann–Whitney, p ≤ 0.001. [file Image2.tiff]

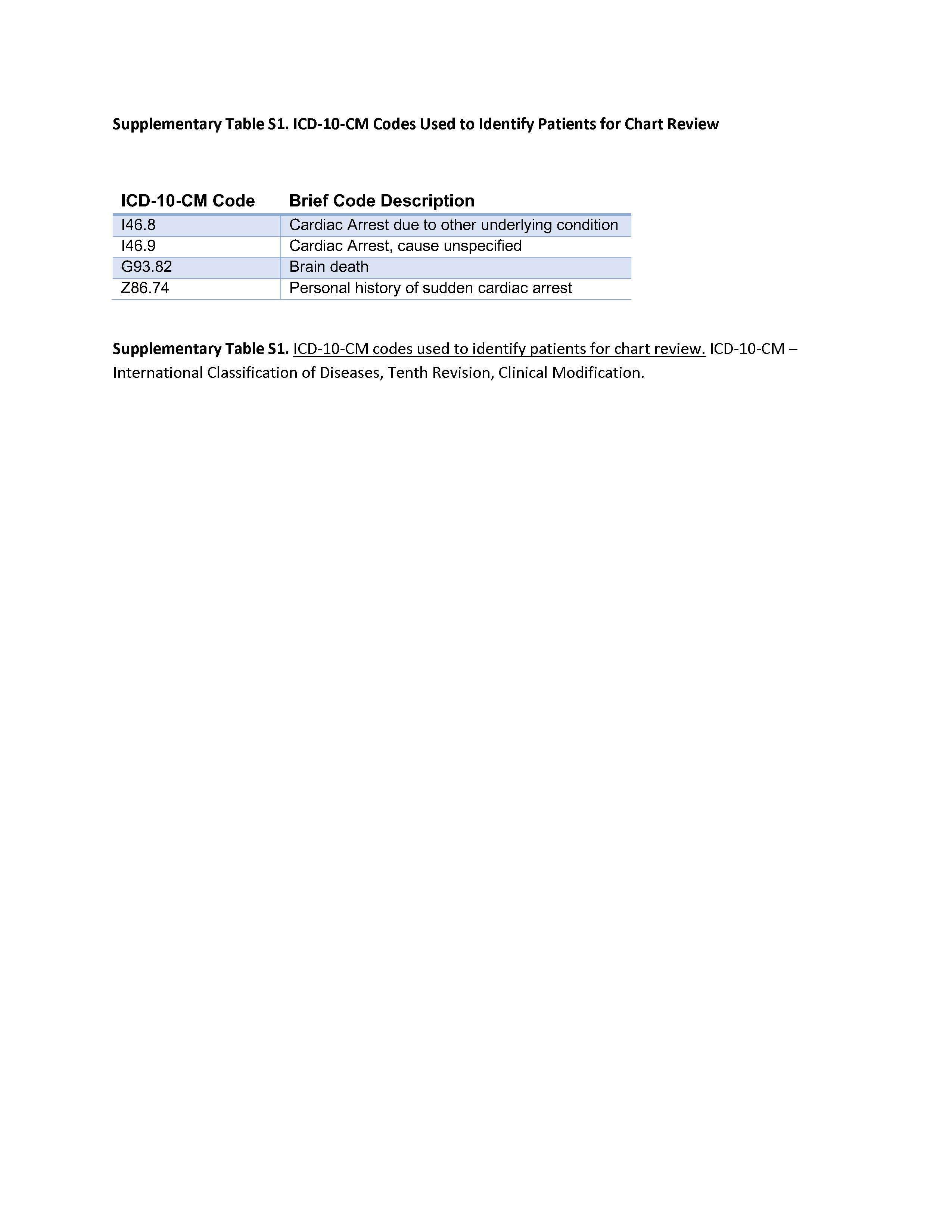

Supplement: Supplementary Table S1 — ICD-10-CM codes used to identify patients for chart review. ICD-10-CM—International Classification of Diseases, Tenth Revision, Clinical Modification. [file Image3.tiff]

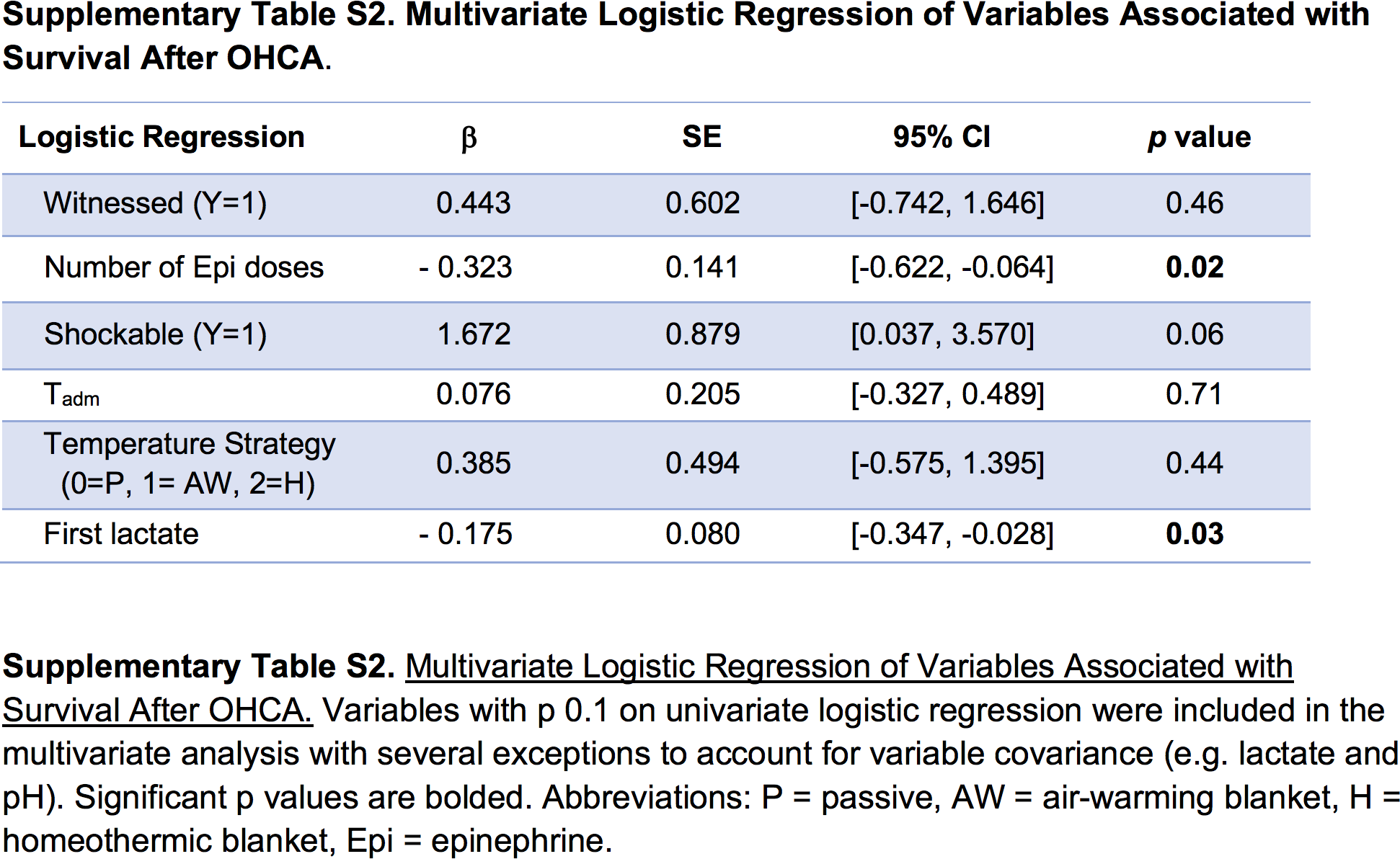

Supplement: Supplementary Table S2 — Multivariate Logistic Regression of Variables Associated with Survival After OHCA. Variables with p ≤ 0.1 on univariate logistic regression were included in the multivariate analysis with several exceptions to account for variable covariance (e.g., lactate and pH). Significant p values are bolded. P, passive; AW, air-warming blanket; H, homeothermic blanket; Epi, epinephrine. [file Image4.tiff]
